# Insurance Does Not Affect Adverse Events While Awaiting Surgery for Ankle Trauma in One System

**DOI:** 10.5811/westjem.2020.5.46861

**Published:** 2020-08-20

**Authors:** Adam B. Dobbins, John Krumme, Monica Gaddis, Shin Hye Park, Manna Varghese, Michael R. Brancato, Christopher M. Shaw, Karen Wambach

**Affiliations:** * Washington Cancer Institute, Department of Musculoskeletal Oncology, Washington DC, Maryland; † University of Missouri Kansas City, Department of Emergency Medicine, Kansas City, Missouri; ‡ University of Missouri Kansas City, Department of Biomedical and Health Informatics, Kansas City, Missouri; § University of Kansas Medical Center, School of Nursing, Department, Kansas City, Kansas; ¶ University of Missouri Kansas City, School of Medicine, Department, Kansas City, Missouri; || University of Missouri Kansas City, Department of Orthopaedic Surgery, Kansas City, Missouri

## Abstract

**Introduction:**

Ankle injuries that are not properly cared for can have devastating effects on a patient’s health and ability to maintain an active lifestyle. Recommended outpatient surgery may be difficult to obtain for many groups of patients, including those without insurance or minority races. Patients who are of low socioeconomic status also have worse outcomes following trauma. The purpose of this study was to examine whether insurance status impacts the number of adverse events that patients face prior to receiving surgical treatment following an emergency department (ED) visit for an acute ankle injury.

**Methods:**

We conducted a retrospective chart review at two medical centers within the same healthcare system. The sample included 192 patients presenting to the ED with an unstable ankle injury between October 1, 2015– May 1, 2018. We used chi-square and t-test analysis to determine differences in rates of adverse events occurring while awaiting surgery.

**Results:**

Few (4%) patients presented as being self-pay. Neither Medicare (χ2 (1) (N = 192) = 2.389, p = .122), Medicaid (χ2 (1), (N = 192) = .084, p = .772), other insurances (χ2 (1) (N = 192) = .567, p = .452), or private insurance (χ2 (1) (N=192) = .000, p = .982) was associated with a difference in rates of adverse events. Likewise, gender (χ2 (1) (N = 192) = .402, p = .526), race (χ2 (3) (N = 192) = 2.504, p = .475), and all other demographic variables failed to show a difference in occurrence of adverse events. Those admitted to the hospital did show a lower rate of adverse events compared to those sent home from the ED (χ2 (1) (N = 192) = 5.452, p = .020). Sampled patients were admitted to the hospital at a high rate (49%).

**Conclusion:**

The sampled facilities did not have adverse event rates that differed based on insurance status or demographic features. These facilities, with hospital-based subsidy programs and higher than expected admission rates, may manage their vulnerable populations well and may indicate their efforts to eliminate health disparity are effective.

## INTRODUCTION

Current standard of care for treatment of unstable ankle fractures in the emergency department (ED) is to evaluate and splint, and then have the patient present for outpatient orthopedic follow-up.^1^ However, those who face barriers to obtaining outpatient treatment may have poorer outcomes than others, indicating this standard of practice may not be optimal for all patients. Navigating outpatient follow-up and outpatient surgery in the face of socioeconomic and payer-source differences may result in significant health disparity in acute ankle-injury patients. Researchers have identified barriers to ED patients getting follow-up. Health systems often do not maintain accurate telephone numbers,^2^ and making follow-up appointments can be difficult or appointments may not be available.^3^ Patients relying on Medicaid or those without insurance^4^,^5^ and minority race populations^6^ have increased difficulty securing follow-up.

Acute ankle injuries can have long-term sequela including recurrent sprains of the injured ankle, instability with sensations of “giving way,” stiffness and swelling, or other symptoms that prevent patients from participating in everyday activities, even with sound treatment.^7^ For individuals who enjoy being active or whose livelihoods depend on standing or moving, failure to return to health following this type of injury can cause significant harm. Along with prolonged instability and potential permanent loss of or decrease in mobility, ankle fractures that do not heal in proper alignment are seven times more likely to develop ankle arthritis, which can cause pain and stiffness requiring long-term treatments.^8^

Trauma patients without insurance have increased rates of mortality and complications,^9^–^11^ indicating that there may be disparities in accessible care for trauma patients. Understanding barriers to proper care may provide information that could lead to achieving more health equality as dictated by Healthy People 2020^12^ and other groups.^13^,^14^ To our knowledge, no studies have looked at follow-up rates or disparities that affect the surgical ankle-fracture patient.

There is a lack of research that explores whether or not the current practice of stabilizing acute ankle injuries in the emergency department (ED) and instructing patients to follow up with a specialist for further evaluation and surgical treatment leads to health disparity among the non-insured. The purpose of this study was to explore whether, among patients who suffer an unstable ankle injury, insurance status is associated with an increased incidence of adverse events experienced prior to surgical correction. Secondary purposes, including whether demographic factors such as gender or race, being homeless, or intoxicated at the time of injury, were also explored.

## METHODS

### Design

We conducted a retrospective chart review with data abstracted from the electronic health records (EHR) at two EDs within a single institution to examine the relationship between payer sources and adverse events while awaiting surgery in patients suffering acute, unstable ankle fractures.

**Population Health Research Capsule**

What do we already know about this issue?

Improperly treated, unstable ankle injuries, which generally require surgical fixation, can lead to a plethora of adverse sequela, including chronic pain and immobility.

What was the research question?

Among patients who suffer an unstable ankle injury, is insurance status associated with an increased incidence of adverse events prior to surgical correction?

What was the major finding of the study?

We found no difference in rates of adverse events prior to obtaining surgery in patients with acute ankle injuries, regardless of insurance type.

How does this improve population health?

This data indicates it is possible to take steps to reduce barriers to optimal surgical treatment of unstable ankle injuries and decrease disparity in healthcare delivery

### Sampling and Setting

We collected data from the EHRs of two EDs within a single health system where the same orthopedic team serves as consultant for both EDs. One ED is an urban, safety-net, non-profit hospital near the downtown area of a large Midwest, US city. It serves as the primary teaching hospital for an adjacent medical college and its mission speaks to providing accessible healthcare regardless of a patient’s ability to pay. As such, it treats many vulnerable populations including the homeless and those without health insurance. The second site lies in a suburban area and focuses on primary care services and provides easy access for acute and well-care needs for all ages. The two facilities share an EHR system. Subsidized care is available at both facilities for qualifying patients who live within the same county as the hospitals and meet income requirements.

*International Classification of Diseases*, 10^th^ edition (ICD-10) codes were identified to capture patients who presented to the EDs with a closed ankle injury for which the standard of care is typically surgical fixation. Table 1 shows a full list of codes used. We obtained all EHRs from patients presenting to either of the two EDs between October 1, 2015–May 1, 2018 and meeting one of the identified ICD-10 codes. We developed a master list of charts that included patient identifying information within a REDCap^T^ database. All study data were collected and managed using REDCap electronic data capture tools.^15^ We assigned random codes to each chart and removed all identifying information. The master list with patient identifying information was stored separately from data collected.

Using predetermined guidelines that indicate surgery would typically be recommended for the treatment of an ankle injury, a fifth-year orthopedic resident reviewed radiographs of each subject to determine whether surgical fixation would likely be recommended. Guidelines for surgical injuries included lateral malleolus injury with joint subluxation, lateral malleolus injury with medial clear space widening on stress or standing view radiograph, displaced medial malleolus fracture, bimalleolar fractures, trimalleolar fractures, or high fibular fractures with a positive stress exam. Those that were determined to be surgical were included. Data were abstracted from the selected charts by two researchers who were blinded to the purpose of the study. We calculated Cohen’s kappa scores to check inter-rater reliability, and the lead researcher trained abstractors to ensure as much consistency between the abstractors as possible.

There were 552 medical records with ankle injuries per the selected ICD codes, of which 255 were identified as unstable after radiograph review. On chart review, 13 were not actually acute ankle injuries or EHR data were not available. An additional 20 patients presented directly to orthopedics or podiatry and were not ED patients, three of whom suffered injuries while hospitalized. For 30 patients, surgery was not recommended, despite their injuries. The most common reasons for not having surgery recommended were co-morbid conditions that increased surgical risks or physician preference at the time of initial evaluation. A sample of 192 cases remained and were included in the study.

### Measures

For this study we considered any ankle injury as found above that is expected to require surgical intervention to promote proper healing as an unstable ankle injury. The dependent variable was adverse events that served as an additional injury or problem with obtaining surgical intervention. Time of surgery served as the time that patient charts were no longer reviewed as they had begun terminal treatment for the injury. Adverse events included the following: re-injury at the original site; delay in surgery greater than three weeks; lost to follow-up where no records up to eight weeks post-injury were found to indicate surgery was ever performed; return ED visits prior to surgery; new traumatic injury; and new pressure ulcer at the site of injury or elsewhere on the body.

The primary independent variable was insurance status and was grouped into the following categories: 1) private insurance; 2) Medicare; 3) Medicaid; 4) worker’s compensation/liability insurance; 5) self-pay/no-charge; and 6) other, for which the majority of “other” patients were included in the hospital-provided subsidy plan. It is important to note that the subsidy plan can be applied retroactively, so many of these patients were likely self-pay at the time of the initial ED visit and retroactively converted to the subsidy plan. Other variables collected were the demographic data of age in years, biological gender, and race/ethnicity grouped as White, Black, Hispanic, or other. Residency information was collected and grouped as private home, nursing home, homeless, or other, and the county and state of residence was included. Alcohol and drug (excluding marijuana) intoxication at the time of injury was collected, identified by healthcare provider notes or a diagnosis code related to alcohol or drug intoxication within the ED chart during the same initial visit for injury.

### Data Analysis

We used SPSS version 22 (IBM Corp, Armonk, NY) for data analysis. All data were imported from REDCap into SPSS for analysis. Prior to collecting data a power analysis identified 196 as a target sample size for this study. Descriptive statistics were examined individually and the chi-square (χ^2^) test of association was applied to categorical independent and dependent variables. The primary independent variable of insurance was examined on each variable in a 2x2 table to determine whether the dependent variable was statistically different when the independent variable of an adverse event occurring was present compared to when not present. We also examined secondary outcomes examined via χ^2^ techniques for categorical data and with the t-test statistic for continuous level data. Significance level was set at less than or equal to .05. We applied Bonferroni adjustments in levels of statistical significance when appropriate after comparing multiple variables against the dependent variable.

### Ethical Considerations

The involved academic institutions’ institutional review boards reviewed all study protocols and permission was granted from the hospital’s privacy committee to use the EHR data. The study was granted exempt classification since only medical records were being used and risk to patients was small. All data were secured within REDCap and patient identifiers were stored separately from the data collected. Patient identifier information was only accessed when it was necessary to review information on the patient within the medical record and used only by researchers tasked with reviewing patient charts. Data collection that involved the use of patient identifying information was always conducted in a private location to prevent possible casual observation of patient information that could occur in a public venue.

## RESULTS

There were 192 patients seen in one of two EDs within this single hospital system who sustained an acute ankle injury that needed surgical repair. The mean age of patients was 43.63 (standard deviation [SD] 14.1) years, and 55% were male. White race was predominant at 46%, with fewer Black (34%), Hispanic (11%), or other (9%) races represented. This reflects a sampling of the general ED population, which was 50% Black, 33% White, and 11% Hispanic. The majority resided in private homes (91%), and approximately 5% were homeless. Fifteen percent were identified as intoxicated with alcohol at the time of initial visit and 5% with other substance intoxication. The ankle injury was an isolated injury in 84% of patients and 49% were admitted to the hospital directly from the ED. Among the 38.3% of patients with “other insurance” listed, almost all had a hospital-specific subsidy applied either at the time of ED visit or applied to their account retroactively. Patients who presented to the ED as self-pay, and had the subsidy applied retroactively, were queried as “other insurance” and did not remain self-pay. Otherwise, insurance classifications were represented as 18.1% with private insurance, 12.4% with Medicare, 16.1% with Medicaid, 10.9% with workers’ compensation or liability insurance, and 4.2% remained self-pay.

Fifteen percent of all patients sustained an adverse event prior to surgical treatment. Related to insurance status, the rate of adverse events ranged from 10% in the workers’ compensation/liability group to 25% in the Medicare group. There were no statistically significant differences in insurance types noted between those with adverse events and those without adverse events.

There were no significant differences in any other demographic variables among those having and not having an adverse event, except for those “not admitted to the hospital” who had a 2.755 increased odds of having an adverse event compared to those admitted directly to the hospital during their initial ED visit (χ^2^_(1)_ (N = 192) = 5.452, p = .020). Reasons for admission were frequently not clear on chart review with 33% of charts not giving any indication of reason for admission. Most, 42%, were admitted by trauma services following a dangerous mechanism of injury, often for observation. Other reasons included 12% admitted due to their comorbidities, 7% for pain control, 4% for social or economic reasons, and 2% for mobility concerns.

Those individuals who sustained multiple injuries at the time of ED visit had 5.814 increased odds of having a presurgical adverse event compared to those having an isolated injury, although this was not statistically significant (χ^2^_(1)_ (N = 192) = 3.613, p = .057). All demographic variables, as well as the results of the comparisons by complication/no complication, are shown in Table 2.

## DISCUSSION

This study, conducted at two EDs within a single hospital system, failed to identify any differences in rates of adverse events prior to obtaining surgery in patients with acute ankle injuries requiring surgical correction regardless of type of insurance coverage. This is in contrast to previous studies in which acute trauma patients had increased rates of mortality and complications when they did not have insurance.^9^–^11^ Furthermore, previous research showed that obtaining follow-up care can be difficult,^2^,^3^ which seems paramount to patients who are often discharged with the intent to secure outpatient surgical services. Previous research also indicated follow-up was particularly difficult to obtain for those on Medicaid and without insurance.^4^–^6^

The current standard of care for ankle fractures such as those focused on in this study is to treat patients on an outpatient basis.^1^ However, among the patients sampled at this facility, nearly half (49%) were admitted to the hospital at the time of their initial ED visit. This is in stark contrast to previously reported admission rates of 17% for ankle fractures in Finland^16^ and 31% in Italy.^17^ Although this facility is a major, inner-city, trauma center, 84% of patients had isolated ankle injuries; thus, severity of illness does not readily explain the high admission rate. Many patients were admitted for observation following a dangerous mechanism of injury. Chart abstraction was attempted to determine the cause of admission for patients; however, in a majority of charts admission decisions were not clear. This facility serves a high volume of patients considered vulnerable; thus, healthcare providers here may be more likely to admit patients for social reasons or to prevent adverse events in comparison to other institutions. Indeed, being admitted at the time of the ED visit was the only statistically significant finding in this study, showing fewer adverse events occurred when patients were directly admitted from the ED.

This healthcare system and the orthopedic group that ultimately makes admission decisions for these patients treat a large number of low-income, racially diverse, and other vulnerable patient populations. These healthcare providers may proactively and aggressively treat these patients, thereby decreasing the odds of the patients receiving disparate care. The orthopedic clinic has also committed to following up with all patients that present through the facilities’ EDs to assist patients to get insurance coverage or hospital-based subsidy, or even making the exception to provide surgery to those who cannot pay. Anecdotally, patients frequently report that other local facilities will not provide them surgical or follow-up services due to their financial/insurance status, despite identifying that their injury needs additional care.

This study also sampled a lower number of self-pay patients than was expected. This study found only about 4% were listed as self-pay compared to national database reports of about 16% in 2010.^18^ This is likely because the institution has a subsidy program. Patients who live within the same county and qualify may obtain reduced or no-cost services despite a lack of insurance. This subsidy program can be applied to ED visits retroactively; thus, a large number of patients who would be self-pay at other facilities were likely marked as “other insurance” in this instance. The EHR does not allow users to separate patients identified initially as self-pay from those who had the subsidy applied after the ED visit. Despite this, neither the remaining self-pay patients nor the “other” insurance category, which includes the subsidy program patients, had a statistically different rate of having adverse events.

Although not statistically significant, Medicare patients had 2.389 increased odds of having an adverse event prior to receiving surgical treatment. This may be a reflection of age-related decreased ability to heal following injury, rather than related to insurance coverage. Patients with an isolated injury had 5.814 decreased odds of having an adverse event. Again, although this finding is not statistically significant, it may suggest that multitrauma patients may be at higher risk than those with isolated ankle injuries.

Currently there is a widespread call to reduce healthcare disparities.^12^–^14^ The findings of this study indicate that this single hospital system may provide appropriate care for vulnerable populations and may be meeting goals to minimize healthcare disparity based on patient insurance status and patient demographics.

## LIMITATIONS

Examination of this data failed to support the primary outcome that insurance status at a single facility correlated with difficulty obtaining surgical correction of an unstable ankle injury. While there were no significant differences, type II error is always a possibility, especially with this small sample size. This study was also limited in its ability to generalize beyond this health system. Given that only a single system was used for data collection in this study, along with the unexpected rates of patients admitted to the hospital from the ED and those with self-pay status, these results may be difficult to extrapolate to any larger population. This may be the result of efforts within this health system to decrease disparity and may well be unlike many other facilities.

This was a retrospective chart review. Data in EHRs are collected by healthcare providers as part of their routine care for patients and are not collected with the methodological rigor that researchers use in collecting data. Therefore, it must be understood that the information gained from these records may contain inaccuracies or information recorded in a way that does not translate well into the research data-collection procedure. Abstractors were trained prior to reviewing charts and were updated if problems arose along the way (eg, properly identifying patients as self-pay or those with hospital-subsidized discount plans); they used standardized forms with precise definitions, and were blinded to the purpose of the study – all methods recommended to strengthen the chart review process.^19^,^20^

The sample of 192 records did not meet the pre-study power estimated need of 196. Including other facilities, or using a national database may help to strengthen future research in this area and provide for increased generalizability. This study was limited in scope by examining only outcomes prior to surgical intervention. Another question of concern to patients would be adverse event occurrence until complete healing of the injury. Factors such as surgical complications, poor wound healing after surgery, hardware failure, and acute or chronic pain are important patient-centered outcomes not examined in this study. The research could also be expanded to include other common, surgically treated fractures such as upper extremity, vertebral, or hip fractures.

## CONCLUSION

This retrospective chart review shows that patients who present to one of two EDs within the same hospital system did not show differences in sustaining adverse events prior to receiving surgical treatment based on insurance status or demographic variables. This is not consistent with other research and may indicate that this facility has implemented progressive policies and procedures to decrease health disparities among patients who fall into vulnerable population categories.

## Figures and Tables

**Table 1:** ICD-10* codes used to capture patients with an unstable ankle injury.

Ankle fracture	Bimalleolar fracture	Lateral malleolus fracture	Medial malleolus fracture	Pilon fracture	Trimalleolar fracture	Distal tibial articular fracture	Syndesmotic injury
S82.843A	S82.841	S82.63XA	S82.53SA	S82.873	S82.851	S82.3	S93.439A
	S82.842	S82.64XA	S82.51XA	S82.871	S82.852	S82.30	S93.431
	S82.843	S82.65XA	S82.52XA	S82.872	S82.853	S82.301	S93.431A
	S82.844	S82.66XA	S82.53XA	S82.873	S82.854	S82.301A	S93.432
	S82.845	S82.61XA	S82.54XA	S82.874	S82.855	S82.302	S93.432A
	S82.846	S82.61XA	S82.55XA	S82.875	S82.856	S82.302A	S93.439
	S82.846	S82.63XA	S82.56XA	S82.876	S82.851A	S82.309	S93.439A
	S82.842A				S82.852A	S82.309A	
	S82.844A				S82.853A	S82.39	
	S82.845A				S82.854A	S82.391	
					S82.855A	S82.391A	
					S82.856A	S82.392	
						S82.392A	
						S82.399	
						S82.399A	

* International Classification of Diseases, 10th edition.

**Table 2:** Demographics and chi-square calculated p-values for subjects with and without adverse events prior to obtaining terminal (surgical) treatment for acute ankle injuries.

Variable	All n(%)	Patients with adverse events n (%)	Patients without adverse events n (%)	χ^2^ statistic	P-value	Odds ratio
Payer source						
Private	35 (18)	5 (15)*	29 (85)	0.000	.982	1.012
Medicare	24 (12)	6 (25)*	18 (75)	2.389	.122	2.212
Medicaid	31 (16)	4 (13)	27 (87)*	0.084	.772	1.182
Workers comp/liability	21 (11)	2 (10)	19 (90)*	0.485	.486	1.704
Self/no pay	8 (4)	2 (15)*	6 (75)	0.727	.394	2.012
Other	74 (38)	9 (12)*	65 (88)	0.567	.452	1.386
Gender						
Male	107 (55)	17 (16)*	89 (84)	0.402	.526	1.302
Female	86 (45)	11 (13)	75 (87)			
Race						
White	88 (46)	12 (14)	76 (86)*	0.117	.732	1.152
Black	66 (34)	12 (18)*	54 (82)	1.045	.307	1.529
Hispanic	21 (11)	1 (5)	20 (95)*	1.826	.177	3.75
Other	17 (9)	3 (18)*	14 (82)	0.141	.708	1.285
Residence						
Private home	176 (91)	24 (14)	151(86)*	1.520	.218	2.111
Nursing home	0					
Homeless	10 (5)	2 (20)*	8 (80)	0.248	.618	1.499
Other	6 (3)	4 (67)*	2 (33)	1.748	.186	3.077
ETOH intoxication						
Yes	29 (15)	3 (10)	26 (90)*	0.493	.483	1.570
No	163 (85)	25 (15)	138 (85)			
Drug intoxication						
Yes	9 (5)	2 (22)*	7 (78)	0.442	.506	1.724
No	183 (95)	26 (14)	157 (86)			
Isolated injury						
Yes	163 (84)	27 (17)	135 (83)*	3.613	.057	5.814
No	30 (16)	1 (3)	29 (97)			
Admitted hospital						
Yes	95 (49)	8 (9)	86 (91)*	5.452	.020**	2.755
No	98 (51)	20 (20)	78 (80)			

	Mean (SD)	Mean (SD)	Mean (SD)	t-test statistic	P-value	

Age#	43.77 (14.0)	44.57(11.8)	43.62 (14.4)	−.330	.742	

Note:

* Higher odds of event occurring,

** Statistically significant with p<.05,

# indicates t-statistic.

*ETOH*, ethyl alcohol; *SD*, standard deviation.
